# Towards pragmatic adaptations: Orthopedic surgery amidst the coronavirus disease 2019 pandemic

**DOI:** 10.1016/j.amsu.2020.11.012

**Published:** 2020-11-06

**Authors:** Maryam Ehtesham, Talal Almas, Muhammad Ali Niaz, Absam Akbar

**Affiliations:** a Royal College of Surgeons in Ireland, Dublin, Ireland; b Aga Khan University, Karachi, Pakistan

## “The crisis you have to worry about most is the one you don’t see coming.”— Mike Mansfield

Since its inception, the coronavirus disease 2019 (COVID-19) has proliferated rapidly, eliciting debilitating ramifications for patients, physicians, and healthcare setups alike. Due to the exorbitant infection-related admissions, healthcare setups have been stretched to their horizons with a resultant depletion of essential resources. Consequently, time-sensitive decisions vouching for the prioritization of infected patients have been made, calling for unprecedented transformations in orthopedic surgical practice. Despite these significant reforms, novel guidelines surrounding the orthopedic surgical milieu remain at the epicenter of an elusive public healthcare conundrum. We hereby delineate these transformations and propose pragmatic strategies to mitigate the concomitant risks.

COVID-19 has elicited widespread repercussions for the surgical specialties. Additional routes of viral entry come entrenched in procedures that necessitate physical contact. Considering this augmented risk of infection, triaging is key. Triaging requires assessing the level of urgency by using urgency scales [1]. Imperatively, the demographic and comorbidities of an individual form the crux of surgical decision-making [1]. Since body fluids act as a nidus for SARS-CoV-2 virions, surgical procedures must vie to reduce the production of aerosols. Orthopedic surgery draws heavily upon high-powered ammunition that generates exorbitant amounts of aerosols. To circumvent this, devices must be operated on the lowest energy setting feasible. Moreover, it is crucial that surgical wards encompass a laminar air flow ventilation system in order to expunge the aerosols. Orthopedic surgeons should stringently adhere to the prescribed personal protective equipment (PPE), including level 4 surgical gowns. Since orthopedic surgery is often laborious, powered air-purifying respirators can be employed in order to circumvent the risks inherent in using standard surgical masks that contain a permeable superior aspect for ventilation. The curation of negative-pressure anterooms can further reduce the risk of transmission. Furthermore, short, succinct procedures should gain precedence over the more complex ones in order to reduce the total operative time.

The cytokine storm (CS) fomented by the severe acute respiratory syndrome coronavirus-2 (SARS-CoV-2) remains a hallmark of severe COVID-19 infection [2]. In elderly and diabetic patients who routinely constitute the demographic most vulnerable to osteoporosis and frailty fractures, COVID-19-induced prothrombotic states can be exacerbated by surgery and subsequent immobilization [3]. Interestingly, stay-at-home measures amidst the pandemic have resulted in an 85% reduction in high-energy fractures [4]. Nevertheless, low-energy fractures due to accidental falls still remain ubiquitous [4]. Furthermore, COVID-19 has been conjectured to adversely impact wound healing [5]. Additionally, in susceptible patients, COVID-19 can potentially spread through uncovered pressure ulcers [5].

The spectrum of urgency of orthopedic surgical procedures ranges from elective procedures to frailty fractures in the elderly. These categories can be divided broadly into mandatory in-patients, non-operative cases, single-day cases, and outpatient clinics. Mandatory in-patients, such as those requiring surgery for hip fractures, must receive expeditious intervention without any pre-operative delays. Contrarily, non-operative cases, especially those with no risk of neurovascular compromise, can potentially be managed conservatively. Trauma clinics can be “virtualized,” with telemedicine playing a pivotal role in facilitating online consultations. Notably, stay-at-home measures have resulted in a significant reduction in high-energy fractures and consequent orthopedic referrals [6]. In its aftermath, a redundancy in the orthopedic staff responsibilities has ensued, paving the path for a novel Rota system that allows a senior clinician to be on the front-line at all times and thus optimizing patient management [6].

Risk stratification remains crucial in the orthopedic setting. Delineating what constitutes an emergent procedure depends not only on the procedure itself, but also on patient characteristics. For instance, a supracondylar humeral fracture, even in a healthy individual, can be associated with neurovascular compromise and should therefore be expeditiously managed. Similarly, articular and periarticular fractures might not be amenable to conservative management. Fundamentals of fixation, including anatomical reduction and rigid fixation, are usually not possible in such instances. In such unavoidable instances, decreasing the cumulative hospital stay and performing surgery only in COVID-free facilities remains the overarching goal. Additionally, the viability of candidates should be assessed by considering a plethora of factors as elucidated by the ASA classification system [7]. In addition to preoperative COVID-19 testing, body temperatures must be monitored on the day of and days preceding the surgical procedure in order to gauge any infective postoperative repercussions.

Amidst the pandemic, the orthopedic surgical milieu finds itself in unprecedented economic duress. In addition to curtailing elective procedures, telemedicine remains pivotal in curbing the associated expenses [8]. Telemedicine facilitates not only expeditious consultations, but also lends to cost-effective measures to combat fractures [8]. In instances where postponing surgery is likely to yield adverse postoperative outcomes, ambulatory surgical facilities can be employed. In a myriad of instances, the categorical absence of a viable technological infrastructure can render the uptake of telemedicine non-pragmatic. In such cases, a newfound predilection towards conservative management—wherever possible—can help optimize the scarce resources.

Over the past 50 years, conservative management has proven itself to be more than just a temporary means of managing fractures. During the first world war, conservative management of open fractures yielded significant reductions in mortality [9]. The principles governing conservative management revolve around fracture reduction and healing by employing casts or splints [9]. Amidst the pandemic, these ostensibly antiquated, yet effective, regimens can enable the restoration of function and range of motion while allowing us to safely defer non-operative surgeries.

It is ironic that despite exorbitant advancements in the orthopedic surgical world over the past century, orthopedic surgery finds itself in a havoc that can be likened to that experienced by Sir Robert Jones in the battlefields of the first world war. Perhaps, instead of embarking on a quest to discover more knowledge, shifting our focus towards optimizing the knowledge we have acquired over centuries through other pandemics, world wars, natural calamities and economic recessions will yield more promising outcomes. Indeed, it will take the unlearning of the connotation of “normalcy” to relearn it as a post-pandemic entity to better inform the debate on what now constitutes an orthopedic “emergency”.

## Provenance and peer-review

Not commissioned, internally reviewed.

## Ethical approval

Due approval from the ethics committee was obtained.

## Source of funding

No funding to declare.

## Author contribution

TA and ME: designing the study and drafting the first version of the manuscript.

MAN and AA: revising the manuscript, and conducting the literature search.

## Registration of research studies

1. Name of the registry:
2. Unique Identifying number or registration ID:
3. Hyperlink to your specific registration (must be publicly accessible and will be checked):

## Guarantor

Talal Almas.

## Declaration of competing interest

None.
